# Circular RNA hsa_circ_0110389 promotes gastric cancer progression through upregulating SORT1 via sponging miR-127-5p and miR-136-5p

**DOI:** 10.1038/s41419-021-03903-5

**Published:** 2021-06-23

**Authors:** Min Liang, Wenxia Yao, Boyun Shi, Xiongjie Zhu, Rui Cai, Zhongjian Yu, Weihong Guo, Huaiming Wang, Zhijie Dong, Mingzhen Lin, Xinke Zhou, Yanfang Zheng

**Affiliations:** 1 Department of Oncology, Guangzhou Key Laboratory of Enhanced Recovery after Abdominal Surgery, The Fifth Affiliated Hospital of Guangzhou Medical University, Guangzhou Medical University, 510700 Guangzhou, China; 2 Medical Oncology Department, Affiliated Cancer Hospital & Institute of Guangzhou Medical University, Guangzhou Medical University, 510095 Guangzhou, China; 3 Department of Center Laboratory, Guangzhou Key Laboratory of Enhanced Recovery after Abdominal Surgery, The Fifth Affiliated Hospital of Guangzhou Medical University, Guangzhou Medical University, 510700 Guangzhou, China; 4 Department of General Surgery, Nanfang Hospital, Southern Medical University, 510515 Guangzhou, China; 5 Department of Gastrointestinal Surgery, The First Affiliated Hospital of Shantou University Medical College, Shantou, China

**Keywords:** Gastrointestinal cancer, Non-coding RNAs

## Abstract

Increasing studies have found that circular RNAs (circRNAs) are aberrantly expressed and play important roles in the occurrence and development of human cancers. However, the function of circRNAs on environmental carcinogen-induced gastric cancer (GC) progression remains poorly elucidated. In the present study, hsa_circ_0110389 was identified as a novel upregulated circRNA in malignant-transformed GC cells through RNA-seq, and subsequent quantitative real-time PCR verified that hsa_circ_0110389 was significantly increased in GC tissues and cells. High hsa_circ_0110389 expression associates with advanced stages of GC and predicts poor prognosis. Knockdown and overexpression assays demonstrated that hsa_circ_0110389 regulates proliferation, migration, and invasion of GC cells in vitro. In addition, hsa_circ_0110389 was identified to sponge both miR-127-5p and miR-136-5p and SORT1 was validated as a direct target of miR-127-5p and miR-136-5p through multiple mechanism assays; moreover, hsa_circ_0110389 sponged miR-127-5p/miR-136-5p to upregulate SORT1 expression and hsa_circ_0110389 promoted GC progression through the miR-127-5p/miR-136-5p–SORT1 pathway. Finally, hsa_circ_0110389 knockdown suppressed GC growth in vivo. Taken together, our findings firstly identify the role of hsa_circ_0110389 in GC progression, which is through miR-127-5p/miR-136-5p–SORT1 pathway, and our study provides novel insight for the identification of diagnostic/prognostic biomarkers and therapeutic targets for GC.

## Introduction

Gastric cancer (GC) is the fifth most common cancer and the third leading cause of cancer-related mortality worldwide [1]. Although the development of therapeutic methods for GC have been greatly improved, the prognosis remains poor and the overall 5-year survival rate is still only <30% [2], mainly because GC is often diagnosed at advanced stage with high metastasis rate and recurrence [3, 4]. The development of GC is considered to be a gradual process and a quantity of risk factors involve in GC etiology, among which environmental carcinogen exposure is one of the most common causes [5]; N-methyl-N′-nitro-N-nitrosoguanidine (MNNG) is a well-known environmental carcinogen that can result in the occurrence of cancers, especially GC [6].

Circular RNAs (circRNAs) are a novel type of single-stranded endogenous noncoding RNAs that are characterized by their covalently continuous loop without 5′ to 3′ polarity and poly (A) tails [7]. Advances in high-throughput RNA sequencing (RNA-seq) technologies and novel bioinformatics algorithms have identified a growing number of circRNAs [8]. And studies have increasingly found that the dynamic expression patterns of circRNAs were involved in a variety of developmental stages, biological processes, and physiological conditions [9, 10]. Multiple kinds of circRNA functions were also found, such as assembling protein complexes [11], modulating the expression of parental genes [12], RNA-protein interaction [13] and functioning as microRNA (miRNA) sponges [14]. In particular, increasing studies have found that circRNAs are dysregulated in human cancers and play important roles in the occurrence and development of human cancers. For example, Liu et al. recently demonstrated that circDLC1 was downregulated in hepatocellular carcinoma (HCC) tissues and inhibits HCC progression via interaction with RNA-binding protein HuR [15]; Zhang et al. previously found that an endogenous circRNA is decreased in glioblastoma and suppresses oncogenic transcriptional elongation through encoding peptides [16]. In the context of GC, although our previous studies have revealed that hsa_circ_0001829 and hsa_circ_006100 participated in the MNNG-induced GC progression [17, 18], the functions and mechanisms of circRNAs on environmental carcinogen-related gastric tumorigenesis remain poorly elucidated.

In this research, we performed RNA-seq analysis to screen differentially expressed circRNAs in MNNG induced malignant-transformed GC cells and identified a novel circRNA hsa_circ_0110389, which is dramatically increased in both GC tissues and cell lines. And high hsa_circ_0110389 expression associates with advanced stages of GC and predicts poor prognosis. Furthermore, knockdown and overexpression results showed that hsa_circ_0110389 regulates GC cells proliferation, migration and invasion in vitro, and animal experiments demonstrated that hsa_circ_0110389 silencing suppresses tumor growth in vivo. Mechanistically, hsa_circ_0110389 functions as a sponge for both miR-127-5p and miR-136-5p to regulate SORT1 expression and consequently promotes GC progression. The present study provides novel insight into the molecular mechanism of carcinogen-induced GC tumorigenesis and further clues for the identification of diagnostic/prognostic biomarkers and therapeutic targets for GC.

## Material and methods

### Tissue collection and ethical approval

A total of 110 pairs of human GC tissues and adjacent normal tissues were surgically obtained from patients with primary GC at Nanfang Hospital (Guangzhou, China) from June 2015 to March 2018. None of the patients received any chemotherapy or radiotherapy before surgery. The clinicopathological features were recorded and confirmed by two independent pathologists according to the guidelines of the Union for International Cancer Control (UICC). Written informed consents were obtained from all patients before enrollment in this study and the study was approved by the Ethics Committee of Nanfang Hospital.

### Cell culture

Normal human gastric epithelial cells-1 (GES-1) and GC cell lines (HGC-27, MKN-45, AGS, SUN-1, NCI-N87, KATO III) were purchased from Type Culture Collection of the Chinese Academy of Science (Shanghai, China). All cells were cultured in RPMI-1640 medium (Gibco) supplemented with 10% fetal bovine serum (FBS; Gibco) and 1% penicillin–streptomycin. Cells were maintained in a humidified incubator at 37 °C containing 5% CO_2_.

### Oligonucleotides and plasmids

Full length hsa_circ_0110389 was cloned into the pCD5-ciR (GENESEED, Guangzhou, China) and the SORT1 gene overexpression vector was cloned into the pcDNA3.0 vector (Promega), and all the sequence was confirmed by Sanger sequencing (Sangon, Shanghai, China). The short hairpin RNAs (shRNAs) for knockdown of hsa_circ_0110389 and SORT1 were purchased from GenePharma (Shanghai, China). The shRNA sequence for hsa_circ_0110389 is 5′-CCUGGAGCUGACCUUU-3′ and for SORT1 is 5′-CCCUCAGAAUUCUGAUUAUUU-3′. The mimics and inhibitors for miR-127-5p and miR-136-5p were purchased from GenePharma (Shanghai, China).

The 3′-untranslated region (3′-UTR) of SORT1 gene containing putative binding sites for miR-127-5p and miR-136-5p was amplified and cloned to the psi-CHECK2 vector (Promega) by Bersinbio Biotechnology Company (Guangzhou, China), named as SORT1-WT. SORT1 gene mutant 3′-UTR recombinant plasmid was generated using the TaKaRa MutanBEST Kit (TaKaRa, Beijing, China), identified as SORT1-Mut.

The full length of hsa_circ_0110389 gene containing putative binding sites for miR-127-5p and miR-136-5p was amplified and cloned to the psi-CHECK2 vector by Bersinbio Biotechnology Company (Guangzhou, China), as CircRNA-WT. Hsa_circ_0110389 mutant recombinant plasmid was generated using the TaKaRa MutanBEST Kit (TaKara, Beijing, China), identified as CircRNA-Mut.

### Cell transfection

GC cells were seeded and incubated for 24 h, and then cells were transfected with oligonucleotides or plasmids using Lipofectamine 2000 (Invitrogen, San Diego, USA) in serum-free medium in accordance with the manufacturer’s instructions for 4–6 h. After incubation, the cellular supernatant was removed and cultured with fresh complete medium for 48 h.

### RNA extraction, nuclear-cytoplasmic fractionation, RNase R treatment and quantitative real-time PCR (qRT-PCR)

TRIzol reagent (Invitrogen) was used to extract total RNA from GC tissues and cells according to the manufacturer’s instructions. Cytoplasmic & Nuclear RNA Purification Kit (Norgen, Canada) was used to extract nuclear and cytoplasmic RNA fractionation following the manufacturer’s protocol. For RNase R treatment, 10 μg total RNA was incubated with 40 U RNase R (Epicentre Technologies, Madison, USA) for 15 min at 37 °C. 2 μg of total RNA was reversely transcribed into complementary DNA (cDNA) using a PrimeScript^™^ RT Master Mix reagent kit (TaKaRa) and used for circRNA and mRNA detection. 2 μg of total RNA was synthesized by the PrimeScript^™^ RT reagent kit (TaKaRa) and used for miRNA detection. Applied Biosystems^™^ PowerUp^™^ SYBR^™^ Green (Thermo Fisher Scientific, Waltham, MA, USA) mix was used for Real-time PCR according to the manufacturer’s protocol. Amplification and detection were performed using Applied Biosystems 7500 Real-time PCR Detection System (ABI, USA). The value of relative circRNA, mRNA or miRNA expression was normalized to GAPDH or U6, respectively. The 2^−△△Ct^ method was used to quantify resulting data. All experiments were performed for three times.

### Western blot

Total proteins from tissues or cell lines were extracted using the RIPA lysis buffer (Beyotime, Shanghai, China). 20 μg proteins were separated in 10% SDS-PAGE and transferred to polyvinylidene difluoride membranes (Roche, Mannheim, Germany), then the transfected membranes were blocked in Tris-buffered saline containing 5% skimmed milk powder for 2 h. Next, the membranes were incubated with Anti-SORT1 (1 μg/ml, ab16640, abcam), Anti-Beclin-1 (1:1000, ab210498, abcam), Anti-LC3B (1:2000, ab192890, abcam) or Anti-p62 (1:1000, ab56416, abcam) at 4 °C overnight. The membranes were then incubated with horseradish peroxidase-conjugated secondary antibodies. Protein signals was visualized by ECL chemiluminescent reagent (Millipore, MA, USA).

### Cell proliferation assay

The cell proliferation was detected using Cell Counting Kit 8 (CCK-8) assay and EdU incorporation assay. For CCK-8 assay, transfected GC cells in logarithmic growth were seeded in 96-well plates cultured for different times. Cell viability was evaluated with CCK-8 kit (Dojindo, Japan) following the manufacturer’s protocol at different time points. At each time point, 10 μl CCK-8 solution was added and incubated for 1 h at 37 °C. The absorbence was measured at 450 nm using Universal Microplate Spectrophotometer (Bio-Tek Instruments, Inc., Winooski, VT, USA). For EdU incorporation assay, GC cell proliferation ability was analyzed using EdU cell proliferation kit (Ribo, Guangzhou, China) according the manufacturer’s protocol as we previously described [18].

### Colony formation assay

GC cells were digested and placed into a 6-well plate at a concentration of 500 cells per well. Then, the cells were cultured routinely for 14 days. Next, the colonies were fixed and stained with 4% paraformaldehyde and 0.1% crystal violet, respectively. Cell colonies were counted and analyzed.

### Transwell migration assay

The in vitro invasion assays were performed using the BD BioCoat Matrigel invasion chambers (Becton Dickinson, NJ, USA) following the manufacturer’s protocol and were performed as previously reported [19].

### Cell wound healing assay

Scratch wound healing assay was performed to detect cell migration ability. The detail methods were performed as previously reported [20]. The cells were counted manually at 0 h and after 48 h post-scratch.

### Fluorescence in situ hybridization (FISH)

The Fluorescence in situ hybridization (FISH) assay was performed as previously reported [14]. In brief, GC cells were seeded and fixed on a coverslip. GC cells were incubated with cy3-labelled hsa_circ_0110389 probe (Ribobio, Guangzhou, China) and FAM-labelled miR-127-5p or miR-136-5p probe (Ribobio, Guangzhou, China) at 37 °C overnight after prehybridization and hybridization. The images were acquired on ZEISS LSM800 Confocal Microscope.

### RNA pull-down assay

The pull-down assay was performed as previously described [21, 22]. Briefly, HGC-27 cells were transfected with Biotinylated miR-127-5p/miR-136-5p or Biotinylated negative control (NC) probe. After 48 h, the cell lysate was obtained and incubated with streptavidin-agarose beads (Thermo Fisher, USA) for 2 h. After the incubation, the bound RNAs were washed and purified for the analysis. Probes were designed and synthesized by Ribobio (Guangzhou, China).

### Luciferase reporter assay

Dual luciferase reporter assay was performed using the procedure as previously described [23]. The relative values of hFluc and hRluc were detected by Centro LB960 XS3 (Berthold, German). Firefly luciferase activity was normalized to Renilla luciferase activity.

### Animal experiments

A total of 24 male BALB/c Nude mice (6 weeks old) were purchased from the animal experiment center of Southern Medical University (Guangzhou, China). The stably cell lines with hsa_circ_0110389 knockdown were constructed with HGC-27 and KATO III cells. 5 × 10^6^ HGC-27 or KATO III cells were dissolved in 100 μl PBS and were subcutaneously injected into the right axilla of each nude mouse. Tumor volumes were measured every 4 days and weights were measured at the final time point; the tumor tissues were then collected for immunostaining analysis. Animal experiments were approved by the Ethics Committee of Southern Medical University.

### Immunohistochemistry (IHC)

IHC was used to measure SORT1 and Ki-67 protein expression in xenografted tumors. Tumors were fixed with 4% paraformaldehyde, embedded in paraffin blocks and sections (4 μm) were prepared. Then, the slides were incubated with the primary antibodies against SORT1 (1:250, ab188586, abcam) or Ki-67 (1:500, ab15580, abcam). The complex was visualized with DAB kit, and the slides were counterstained with hematoxylin.

### Statistical analysis

Data are expressed as mean ± standard deviation (SD) and all experiments were performed at least three times. Data were calculated by the Fisher’s exact test or *χ*^2^. Pearson’s test (r, *P*) was used for the correlation analysis. The paired or unpaired Student’s *t* test was used to compare mean values between two samples and One-way ANOVA was used for multi-group comparisons of mean values. The survival curves were drawn using the Kaplan–Meier method and were analyzed by log-rank tests. Univariate analysis and multivariate models were performed with a Cox proportional hazards regression model. The SPSS 20.0 software was used for statistical analysis. *P* < 0.05 was considered statistical significance.

## Results

### Hsa_circ_0110389 is up-regulated in GC tissues and cells, and high hsa_circ_0110389 expression associates with advanced stages of GC and predicts poor prognosis

**Fig. 1:**
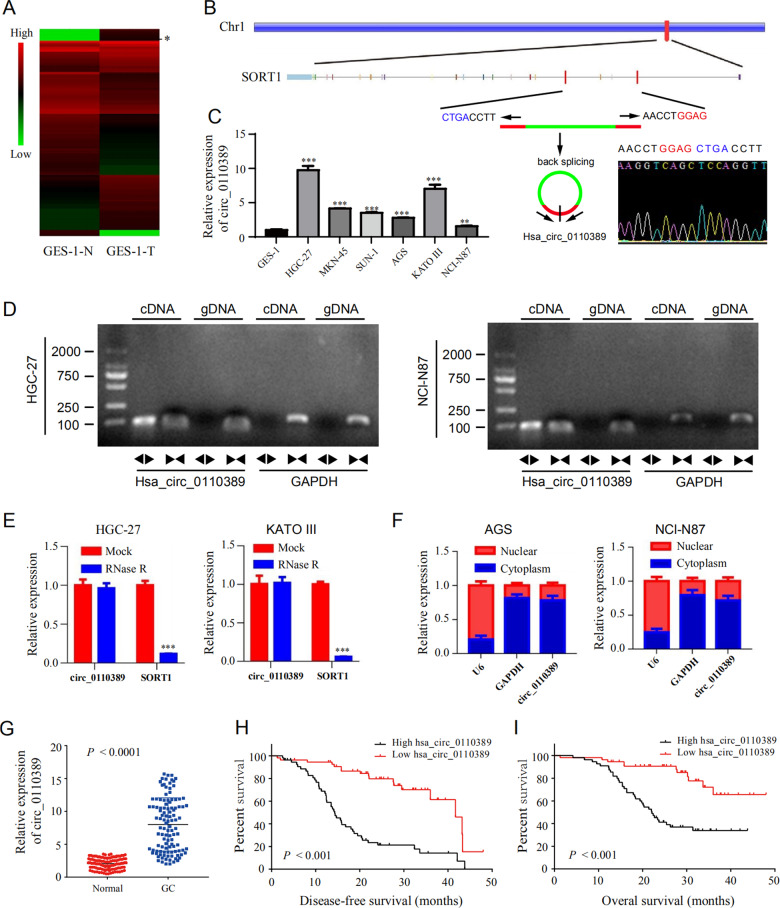
Hsa_circ_0110389 is up-regulated in gastric cancer tissues and predicts poor prognosis. **A** Heat map of RNA-seq analysis between GES-1-T and GES-1-N cells. The asterisk indicates hsa_circ_0110389. **B** Schematic illustration of hsa_circ_0110389 formation via the circularization of exons in SORT1 gene. Sanger sequencing conformed the head-to-tail splicing of hsa_circ_0110389. **C** Expression of hsa_circ_0110389 in different cell lines. **D** The gel electrophoresis validated the existence of hsa_circ_0110389. Divergent primers amplified hsa_circ_0110389 in cDNA but not in gDNA. GAPDH was used as a linear control. **E** Expression of hsa_circ_0110389 and SORT1 mRNA in both HGC-27 and KATO III cells was detected by qRT-PCR in the presence or absence of RNase R. **F** Hsa_circ_0110389 was mainly located in the cytoplasm by nuclear-cytoplasmic fractionation assay. **G** Relative expression of hsa_circ_0110389 in 110 paired GC tissues and adjacent normal tissues. **H**, **I** Kaplan–Meier survival analysis (log-rank test) showed that GC patients with high hsa_circ_0110389 expressions have a lower disease-free survival and overall survival than these with low hsa_circ_0110389 expressions (*P* < 0.05). All data are presented as mean ± SD. ***P < 0.001.

RNA-seq analysis between malignant-transformed human gastric epithelial cells (GES-1-T cells) induced by MNNG and NC cells (GES-1-N cells) was conducted in our previous study [17, 18], and the heat map expression results showed that hsa_circ_0110389, accession id according to the annotation of circBase (http://www.circbase.org/), was highly up-regulated in GES-1-T cells (Fig. 1A). With a spliced mature sequence length of 273 bp, hsa_circ_0110389 is derived from SORT1 gene locus and is located at chr1:109884635-109888500; sanger sequencing confirmed the head-to-tail splicing of hsa_circ_0110389 (Fig. 1B). We then validated the induced up-regulation of hsa_circ_0110389 in GC cells by qRT-PCR assay; consistent with the RNA-seq results, hsa_circ_0110389 showed increased expression in all the six GC cell lines than in GES-1 cell (Fig. 1C), and among the GC cell lines, HGC-27 and KATO III cells exhibited the highest expression level of hsa_circ_0110389 while AGS and NCI-N87 cells demonstrated the lowest expression level (Fig. 1C). Then, divergent and convergent PCR primers were designed to validate the backsplicing formation of hsa_circ_0110389 using the total RNA and genomic DNA from HGC-27 and KATO III cells. As expected, the gel electrophoresis results showed that backsplicing junctions of hsa_circ_0110389 using divergent primers were amplified only in cDNA samples but not in genomic DNA samples (Fig. 1D). Furthermore, HGC-27 and KATO III cells were treated with RNase R to evaluate the stability of hsa_circ_0110389; as shown in Fig. 1E, hsa_circ_0110389 resisted the digestion of RNase R whereas linear of SORT1 was digested sharply, indicating that hsa_circ_0110389 has a closed loop. In addition, the results of nuclear and cytoplasmic fractionation demonstrated that hsa_circ_0110389 was predominantly localized in the cytoplasm (Fig. 1F).

To further investigate the expression levels of hsa_circ_0110389 in GC tissues, we collected 110 pairs of fresh frozen GC tissues and adjacent normal tissues. The qRT-PCR results showed that hsa_circ_0110389 expression was upregulated in GC tissues compared with normal tissues (Fig. 1G). Then, we evaluated the association between the hsa_circ_0110389 expression level and different clinicopathological characteristics of 110 patients with GC. Based on the median expression of hsa_circ_0110389, patients were divided into two groups (high hsa_circ_0110389 group and low hsa_circ_0110389 group). As shown in Table S1, high expression of hsa_circ_0110389 was associated with poor tumor differentiation and advanced pathological T stage, N stage, M stage and UICC stage (*P* < 0.05); no significant differences were observed in the other clinical and pathological characteristics between the high- and low-hsa_circ_0110389 groups. In addition, the Kaplan–Meier analysis results showed that GC patients with high hsa_circ_0110389 expression had a significantly shorter overall survival (OS) and disease-free survival (DFS) than those with low hsa_circ_0110389 expression (Fig. 1H-I), which suggested that hsa_circ_0110389 could serve as a prognostic marker. Moreover, to assess whether the ability of hsa_circ_0110389 to predict survival is independent of other clinical or pathological factors of GC patients, univariate and multivariate Cox proportional hazards analyses were performed. According to the results, in addition to T stage, N stage, M stage and UICC stage, high expression level of hsa_circ_0110389 was a significant predictor of poor prognosis in GC (*P* < 0.001 for both OS and DFS) (Table 1). And the results showed that hsa_circ_0110389 expression level and UICC stage were independent prognostic factors in GC patients (Table 1).

**Table 1:** Multivariate analysis identifies factors influencing the overall survival rate and disease-free survival rate of gastric cancer patients.

	Overall survival duration				Disease-free survival duration			
Factors	Univariate analysis		Multivariate analysis		Univariate analysis		Multivariate analysis	
	HR (95% CI)	*P* value	HR (95% CI)	*P* value	HR (95% CI)	*P* value	HR (95% CI)	*P* value
Age	0.976 (0.536–1.777)	0.937			0.742 (0.432–1.273)	0.279		
Gender	1.063 (0.589–1.916)	0.840			0.999 (0.591–1.688)	0.996		
Differentiation	1.536 (0.828–2.847)	0.173			1.413 (0.834–2.393)	0.199		
T stage	2.580 (1.203–5.536)	0.015	0.404 (0.091–1.791)	0.233	3.179 (1.647–6.139)	0.001	0.971 (0.288–3.272)	0.962
N stage	3.456 (1.610–7.417)	0.001	1.075 (0.387–2.988)	0.890	3.923 (2.076–7.415)	0.000	1.096 (0.460–2.615)	0.836
M stage	2.347 (1.293–4.261)	0.005	1.076 (0.547–2.116)	0.833	2.154 (1.249–3.713)	0.006	0.782 (0.422–1.450)	0.435
UICC stage	4.284 (1.912–9.599)	0.000	5.480 (1.085–2.672)	0.039	5.335 (2.595–10.968)	0.000	3.963 (1.032–15.214)	0.045
Nerve invasion	0.830 (0.465–1.480)	0.528			0.996 (0.592–1.675)	0.988		
Vessel invasion	0.990 (0.549–1.785)	0.974			0.997 (0.594–1.674)	0.990		
hsa_circ_0110389 expression	4.361 (2.204–8.628)	0.000	3.061 (1.207–7.763)	0.018	4.739 (2.684–8.366)	0.000	3.375 (1.549–7.354)	0.002

Collectively, these observations indicate that hsa_circ_0110389 is increased in GC cell lines and tissues, and hsa_circ_0110389 expression level is an independent prognostic marker for survival of GC patients.

### Hsa_circ_0110389 promotes proliferation, migration and invasion of GC cells in vitro

**Fig. 2:**
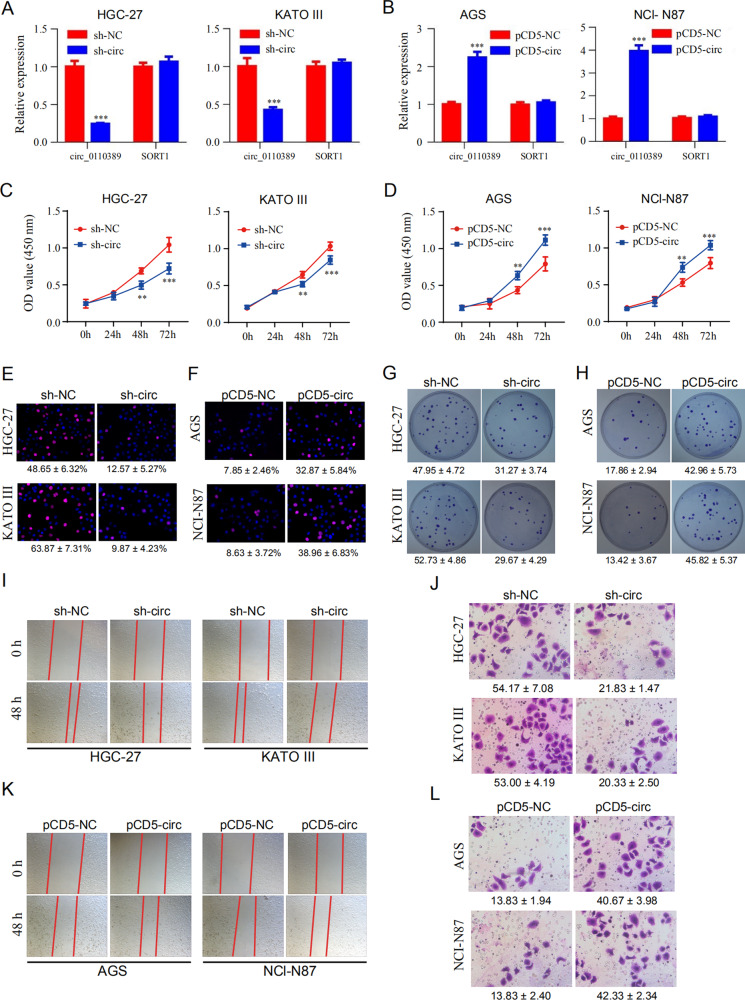
Hsa_circ_0110389 promotes the proliferation, migration and invasion of GC cells in vitro. **A** Expression of hsa_circ_0110389 was significantly decreased in HGC-27 and KATO III cells after transfection of sh-hsa_circ_0110389. **B** Expression of hsa_circ_0110389 was significantly increased in AGS and NCI-N87 cells after transfection of hsa_circ_0110389 overexpression plasmids. **C** CCK-8 results showed that knockdown of hsa_circ_0110389 suppressed HGC-27 and KATO III cell proliferation. **D** CCK-8 results showed that overexpression of hsa_circ_0110389 promoted AGS and NCI-N87 cell proliferation. **E** EdU staining results showed that knockdown of hsa_circ_0110389 suppressed HGC-27 and KATO III cell proliferation. **F** EdU staining results showed that overexpression of hsa_circ_0110389 promoted AGS and NCI-N87 cell proliferation. **G** Colony formation results showed that knockdown of hsa_circ_0110389 suppressed HGC-27 and KATO III cell growth in vitro. **H** Colony formation results showed that overexpression of hsa_circ_0110389 promoted AGS and NCI-N87 cell growth in vitro. **I** Wound healing assay of HGC-27 and KATO III cells transfected with sh-hsa_circ_0110389 or control. **J** Wound healing assay of AGS and NCI-N87 cells transfected with hsa_circ_0110389 overexpression or control plasmids. **K** Transwell-invasion assay of HGC-27 and KATO III cells transfected with sh-hsa_circ_0110389 or control. **L** Transwell-invasion assay of AGS and NCI-N87 cells transfected with hsa_circ_0110389 overexpression or control plasmids. All data are presented as mean ± SD. **P < 0.01, ***P < 0.001.

To evaluate the biological functions of hsa_circ_0110389 in GC cells, HGC-27 and KATO III cells were transfected with shRNA which targeted the junction sites of hsa_circ_0110389, and AGS and NCI-N87 cells were transfected with overexpression plasmids of hsa_circ_0110389. The expression of hsa_circ_0110389 was significantly decreased in HGC-27 and KATO III cells after transfected with shRNA, while SORT1 mRNA did not change (Fig. 2A). Similarly, the expression of hsa_circ_0110389 was obviously increased in AGS and NCI-N87 cells after transfected with overexpression plasmids whereas no change in SORT1 mRNA was observed (Fig. 2B). Then CCK-8 and EdU assays were performed to analyze the cell proliferation; as shown, knockdown of hsa_circ_0110389 suppressed HGC-27 and KATO III cell proliferation, while overexpression of hsa_circ_0110389 promoted AGS and NCI-N87 cell proliferation (Fig. 2C-F; Fig. S1A-B). Furthermore, colony-forming ability of HGC-27 and KATO III cell lines were significantly reduced after knockdown of hsa_circ_0110389, and colony-forming ability of AGS and NCI-N87 cell lines were significantly reduced after overexpression of hsa_circ_0110389 (Fig. 2G-H; Fig. S1C-D).

In addition, wound scratch assay and transwell-invasion assay results showed that the mobility of GC cells was significantly decreased by knockdown of hsa_circ_0110389 in HGC-27 and KATO III cells (Fig. 2I and J; Fig. S2A and C). Furthermore, overexpression of hsa_circ_0110389 promoted the invasion and migration of AGS and NCI-N87 cells (Fig. 2K and L; Fig. S2B and D).

Altogether, these results strongly suggest that hsa_circ_0110389 regulates proliferation, migration and invasion of GC cells in vitro.

### Hsa_circ_0110389 acts as a molecular sponge for miR-127-5p and miR-136-5p

**Fig. 3:**
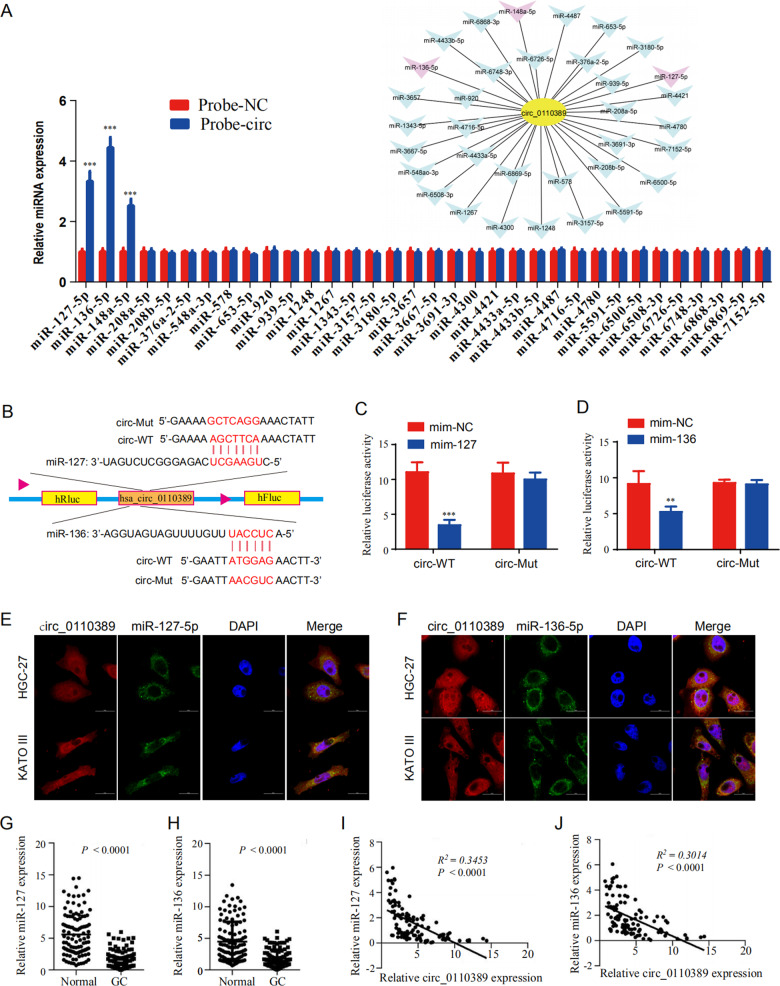
Hsa_circ_0110389 acts as a molecular sponge for miR-127-5p and miR-136-5p. **A** Bioinformatics analysis showed potential binding interaction between hsa_circ_0110389 and different miRNAs, and RNA pull-down results showed that hsa_circ_0110389 may directly bind with miR-127-5p, miR-136-5p and miR-148a-5p. **B** Schematic illustration of the sequences of wild type hsa_circ_0110389 (circ-WT) and mutant hsa_circ_0110389 (circ-Mut) in the binding sites of miR-127-5p and miR-136-5p. **C**, **D** Relative luciferase activities were detected in GC cells after transfecting luciferase reporter plasmids with wild type or mutant of hsa_circ_0110389 with miR-127-5p/miR-136-5p or negative control. mim, mimic. **E**, **F** FISH for subcellular localization of hsa_circ_0110389 and miR-127-5p or miR-136-5p in HGC-27 and KATO III cells. **G**, **H** Expression of miR-127-5p (**G**) or miR-136-5p (**H**) in 110 paired GC tissues and adjacent normal tissues. **I**, **J** Correlation between miR-127-5p (**I**) or miR-136-5p (**J**) and hsa_circ_0110389 expression in 110 paired GC tissues. The correlation was measured by Pearson correlation analysis. All data are presented as mean ± SD. **P < 0.01, ***P < 0.001.

Considering that circRNAs localized in the cytoplasm have been universally reported to function as miRNA sponges, we then investigated whether hsa_circ_0110389, mainly distributed in the cytoplasm of GC cells (Fig. 1F), could act as a miRNA sponge. We performed bioinformatics analysis to predict the potential sponging miRNAs of hsa_circ_0110389, and 34 miRNAs were predicted to potentially bind to hsa_circ_0110389; among these candidate miRNAs, miR-127-5p, miR-136-5p and miR-148a-5p were demonstrated to directly bind with hsa_circ_0110389 by the RNA pull-down assays (Fig. 3A). For further investigation, we concentrated on two miRNAs, miR-127-5p and miR-136-5p, which showed stronger binding ability than miR-148a-5p (Fig. 3A). The potential binding sites of miR-127-5p and miR-136-5p within hsa_circ_0110389 predicted by starBase [24] were demonstrated in Fig. 3B. And the binding ability between miR-127-5p/miR-136-5p and hsa_circ_0110389 were further confirmed by dual luciferase reporter assays; results demonstrated that co-transfection of circRNA-WT and miR-127-5p/miR-136-5p mimic significantly decreased the luciferase activity, whereas co-transfection of circRNA-Mut and miR-127-5p/miR-136-5p mimic did not affect the luciferase activity (Fig. 3C-D). In addition, FISH results showed that hsa_circ_0110389 and miR-127-5p or miR-136-5p were co-localized predominantly in the cytoplasm of HGC-27 and KATO III cells (Fig. 3E-F).

Furthermore, we detected the expression levels of miR-127-5p and miR-136-5p in GC tissues and adjacent normal tissues, the qRT-PCR results showed that the expression levels of both miR-127-5p and miR-136-5p were significantly decreased in GC tissues compared with normal tissues (Fig. 3G-H). Moreover, results demonstrated that the expression levels of miR-127-5p and miR-136-5p were negatively correlated with the expression of hsa_circ_0110389 (Fig. 3I-J).

These observations collectively indicate that hsa_circ_0110389 directly binds to miR-127-5p and miR-136-5p, acting as a sponge for miR-127-5p and miR-136-5p.

### Hsa_circ_0110389 promotes proliferation, migration and invasion of GC cells via miR-127-5p and miR-136-5p

**Fig. 4:**
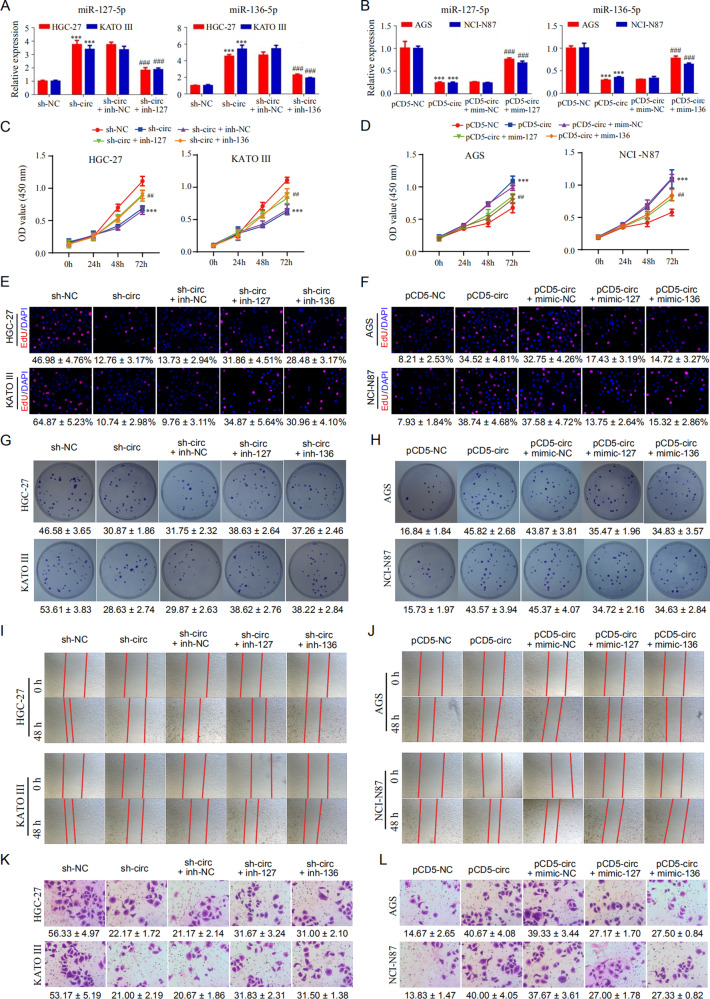
Hsa_circ_0110389 promotes the proliferation, migration and invasion of GC cells via miR-127-5p and miR-136-5p. **A** The expression of miR-127-5p or miR-136-5p was significantly increased in HGC-27 and KATO III cells after transfected with hsa_circ_0110389 shRNA and then decreased when HGC-27 and KATO III cells were co-transfected with miR-127-5p or miR-136-5p inhibitors. **B** The expression of miR-127-5p or miR-136-5p was obviously decreased in AGS and NC-N87 cells after transfected with hsa_circ_0110389 overexpression plasmids and then increased when AGS and NC-N87 cells were co-transfected with miR-127-5p or miR-136-5p mimics. **C**, **E**, **G** miR-127-5p or miR-136-5p inhibitors effectively reversed the hsa_circ_0110389 knockdown induced suppression of proliferation and growth in HGC-27 and KATO III cells by CCK-8 assay (**C**), EdU staining (**E**) or colony formation assay (**G**). **D**, **F**, **H** miR-127-5p or miR-136-5p mimics effectively reversed the hsa_circ_0110389 overexpression induced promotion of proliferation in AGS and NCI-N87 cells by CCK-8 assay (**D**), EdU staining (**F**) or colony formation assay (**H**). *vs sh-NC/pCD5-NC group, #vs inh-NC/mim-NC group. **I** Wound healing results showed that the promoting effect of hsa_circ_0110389 knockdown on migration of HGC-27 and KATO III cells was blocked by miR-127-5p or miR-136-5p inhibitors. **J** Wound healing results showed that the suppressed role of hsa_circ_0110389 overexpression in migration of AGS and NCI-N87 cells was reversed by miR-127-5p or miR-136-5p mimics. **K** Transwell assay results showed that the suppressed role of hsa_circ_0110389 silencing in invasion of HGC-27 and KATO III cells was reversed by miR-127-5p or miR-136-5p inhibitors. **L** Transwell assay results showed that the promoting effect of hsa_circ_0110389 overexpression on invasion of AGS and NCI-N87 cells was blocked by miR-127-5p or miR-136-5p mimics. inh inhibitor, mim mimic. *vs sh-NC/pCD5-NC group, #vs inh-NC/mim-NC group. All data are presented as mean ± SD. **p < 0.01, ***p < 0.001, ^##^*p* < 0.01, ^###^*p* < 0.001.

To investigate whether hsa_circ_0110389 regulates GC progression by sponging miR-127-5p and miR-136-5p, HGC-27 and KATO III cells were co-transfected with hsa_circ_0110389 shRNA and miR-127-5p or miR-136-5p inhibitors, and AGS and NCI-N87 cells were co-transfected with hsa_circ_0110389 overexpression plasmids and mimics of miR-127-5p or miR-136-5p. The expression of miR-127-5p or miR-136-5p was significantly increased in HGC-27 and KATO III cells after transfected with hsa_circ_0110389 shRNA and then decreased when HGC-27 and KATO III cells were co-transfected with miR-127-5p or miR-136-5p inhibitors (Fig. 4A). Similarly, the expression of miR-127-5p or miR-136-5p was obviously decreased in AGS and NC-N87 cells after transfected with hsa_circ_0110389 overexpression plasmids and then increased when AGS and NC-N87 cells were co-transfected with miR-127-5p or miR-136-5p mimics (Fig. 4B). Subsequent CCK-8 assay, EdU staining and clone formation assay revealed that inhibitors of miR-127-5p or miR-136-5p significantly reversed the hsa_circ_0110389 knockdown induced suppression of proliferation in HGC-27 and KATO III cells (Fig. 4C, E and G; Fig. S1E and G) and miR-127-5p or miR-136-5p mimics effectively reversed the hsa_circ_0110389 overexpression induced promotion of proliferation in AGS and NCI-N87 cells (Fig. 4D, F and H; Fig. S1F and H).

In addition, transwell and wound healing assays showed that the suppressed effect of hsa_circ_0110389 silencing on invasion and migration of HGC-27 and KATO III cells was reversed by miR-127-5p or miR-136-5p inhibitors (Fig. 4I and K; Fig. S2E and G), and likewise the promoting role of hsa_circ_0110389 overexpression in invasion and migration of AGS and NCI-N87 cells was blocked by miR-127-5p or miR-136-5p mimics (Fig. 4J and L; Fig. S2 F and H).

All these findings indicated that hsa_circ_0110389 promotes proliferation, migration and invasion of GC cells through sponging miR-127-5p and miR-136-5p.

### SORT1 is validated as a target gene of miR-127-5p and miR-136-5p

**Fig. 5:**
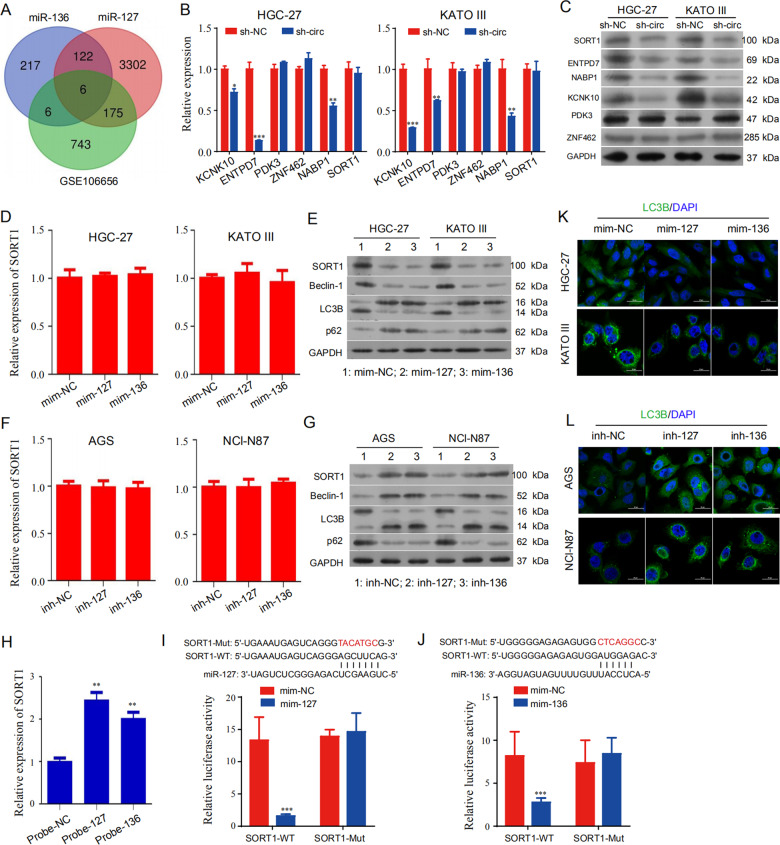
SORT1 is a target gene of miR-127-5p and miR-136-5p. **A** Computational prediction with Gene Expression Omnibus (GEO, GSE106656) and TargetScan to identify the overlapped potential target genes of miR-127-5p and miR-136-5p. **B**, **C** The mRNA and protein expression levels of six overlapped target genes in HGC-27 and KATO III cells with hsa_circ_0110389 knockdown by qRT-PCR and western blot. **D** The mRNA level of SORT1 in HGC-27 and KATO III cells with miR-127-5p or miR-136-5p mimics by qRT-PCR. **E** The protein levels of SORT1 and autophagy-related genes in HGC-27 and KATO III cells with miR-127-5p or miR-136-5p mimics by western blot. **F** The mRNA level of SORT1 in AGS and NCI-N87 cells with miR-127-5p or miR-136-5p inhibitors by qRT-PCR. **G** The protein levels of SORT1 and autophagy-related genes in AGS and NCI-N87 cells with miR-127-5p or miR-136-5p inhibitors by western blot. **H** RNA pull-down results showed that miR-127-5p and miR-136-5p may directly bind with SORT1. **I**, **J** (Up) The binding sites of wild type or mutant SORT1 3′-UTR with miR-127-5p (**I**) or miR-136-5p (**J**). (Down) Dual luciferase reporter assays demonstrated that SORT1 is a direct target of miR-127-5p (**I**) or miR-136-5p (**J**). **K** The expression of LC3B in HGC-27 and KATO III cells with miR-127-5p or miR-136-5p mimics by IF analysis. **L** The expression of LC3B in AGS and NCI-N87 cells with miR-127-5p or miR-136-5p inhibitors by IF analysis. All data are presented as mean ± SD. *p < 0.05, ** *p* < 0.01, ***p < 0.001.

We then utilized TargetScan [25] prediction and Gene Expression Omnibus (GEO) dataset (GSE106656) to identify the potential target genes of miR-127-5p and miR-136-5p, and six overlapped potential genes were identified, those were KCNK10, ENTPD7, NABP1, PDK3, ZNF462 and SORT1 (Fig. 5A). qRT-PCR results showed that hsa_circ_0110389 knockdown inhibited *KCNK10*, *ENTPD7* and *NABP1* mRNA expression with no effect on the mRNA expressions of *PDK3*, *ZNF462* and *SORT1* genes (Fig. 5B). Western blot results showed that hsa_circ_0110389 knockdown suppressed SORT1, KCNK10, ENTPD7 and NABP1 protein expression with no effect on the protein expressions of PDK3 and ZNF462 (Fig. 5C). Interestingly, hsa_circ_0110389 silencing was shown to cause inhibition of SORT1 protein level without a reduction in SORT1 mRNA level; since miRNAs inhibit target protein synthesis either by suppressing translation and/or by degrading of mRNA targets through inducing deadenylation [26], the above results support a hypothesis that the sponging miRNAs by hsa_circ_0110389 could inhibit translation of target SORT1 without effect on SORT1 mRNA degradation. Subsequent results also support this hypothesis. qRT-PCR and Western blot results showed that miR-127-5p or miR-136-5p mimics suppressed SORT1 protein expression and miR-127-5p or miR-136-5p inhibitors increased SORT1 protein expression (Fig. 5E and G), and simultaneously neither miR-127-5p nor miR-136-5p could affect SORT1 mRNA level (Fig. 5D and F).

Further assays were conducted to confirm the interaction between miR-127-5p/miR-136-5p and SORT1. RNA pull-down results showed that both miR-127-5p and miR-136-5p could bind with SORT1 (Fig. 5H). Moreover, we performed dual luciferase reporter experiments and results demonstrated that cells co-transfected with the vectors containing 3′-UTR-WT regions of SORT1 (SORT1-WT) and miR-127-5p/miR-136-5p mimics had significantly less luciferase activity than the controls, whereas co-transfection with the vectors containing mutant 3′-UTR regions of SORT1 (SORT1-Mut) blocked this effect (Fig. 5I-J).

In addition, besides decreased protein expression of SORT1, HGC-27 and KATO III cells transfected with miR-127-5p or miR-136-5p mimics simultaneously demonstrated decreased protein expression of Beclin-1 and LC3B II/I and increased protein expression of p62 (Fig. 5E), which suggested suppressed autophagy. In contrast, aside from increased expression of SORT1, AGS and NCI-N87 cells transfected with miR-127-5p or miR-136-5p inhibitors also showed increased expression of Beclin-1 and LC3B II/I and decreased expression of p62 (Fig. 5G), which indicated stimulated autophagy. Consistently, IF assay by anti-LC3B staining showed that HGC-27 and KATO III cells transfected with miR-127-5p or miR-136-5p mimics demonstrated decreased expression of LC3B II/I (Fig. 5K), while AGS and NCI-N87 cells transfected with miR-127-5p or miR-136-5p inhibitors showed increased expression of LC3B II/I (Fig. 5L).

Taken together, these results indicated that SOTR1 is a target gene of both miR-127-5p and miR-136-5p.

### Hsa_circ_0110389 promotes proliferation, migration and invasion of GC cells through SORT1

**Fig. 6:**
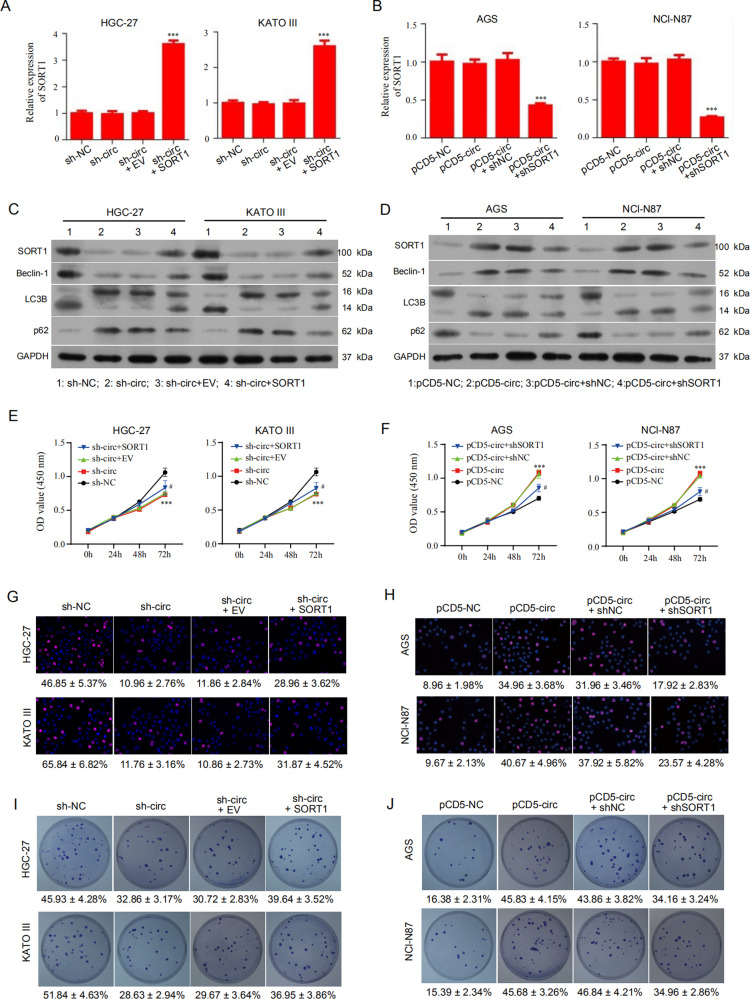
Hsa_circ_0110389 promotes the proliferation of GC cells through SORT1. **A**, **C**, **E**, **G**, **I** HGC-27 and KATO III cells were transfected with sh-NC, sh-circ, sh-circ+EV or sh-circ+SORT1. Then the relative mRNA and protein expression of SORT1 was respectively measured by qRT-PCR analysis (**A**) and western blot assays (**C**), and the ability of cell proliferation and growth was assessed by CCK-8 assay (**E**), EdU assay (**G**) or colony formation assay (**I**). **B**, **D**, **F**, **H**, **J** AGS and NCI-N87 cells were transfected with pCD5-NC, pCD5-circ, pCD5-circ+shNC or pCD5-circ+shSORT1. Then the relative mRNA and protein expression of SORT1 was respectively measured by qRT-PCR analysis (**B**) and western blot assays (**D**), and the ability of cell proliferation and growth was assessed by CCK-8 assay (**F**), EdU assay (**H**) or colony formation assay (**J**). **C**, **D** The protein levels of autophagy-related genes (Beclin-1, LC3B and p62) were also analyzed by western blot assays. All data are presented as mean ± SD. EV empty vector. *vs sh-NC/pCD5-NC group, #vs EV/shNC group. **p < 0.01, ***p < 0.001, ^##^*p* < 0.01, ^###^*p* < 0.001.

To investigate whether hsa_circ_0110389 promotes GC progression through SORT1, HGC-27 and KATO III cells were co-transfected with hsa_circ_0110389 shRNA and SORT1 overexpression vector, and AGS and NCI-N87 cells were co-transfected with hsa_circ_0110389 overexpression vector and SORT1 shRNA. qRT-PCR results showed that knockdown or overexpression of hsa_circ_0110389 did not affect SORT1 mRNA expression, which is consistent with the results of Fig. 5B; however, overexpression or knockdown of SORT1 did affect the mRNA level of itself (Fig. 6A-B). Further Western blot results showed that the suppressed role of hsa_circ_0110389 shRNA in SORT1 expression was reversed by SORT1 overexpression (Fig. 6C) and the promoting effect of hsa_circ_0110389 overexpression on SORT1 expression was blocked by SORT1 shRNA (Fig. 6D). Subsequent CCK-8, EdU staining, clone formation, transwell and wound healing assays showed that SORT1 overexpression significantly reversed the hsa_circ_0110389 knockdown induced suppression of proliferation, invasion and migration in HGC-27 and KATO III cells (Fig. 6E, G and I; Fig. S1I and K; Fig. S3A, C, E and G) and SORT1 knockdown significantly blocked the hsa_circ_0110389 overexpression induced promotion of proliferation, invasion and migration in AGS and NCI-N87 cells (Fig. 6F, H and J; Fig. S1J and L; Fig. S3B, D, F and H). Collectively, these results indicated that hsa_circ_0110389 promotes proliferation, migration, and invasion of GC cells through SORT1.

In addition, Western blot results showed that knockdown of hsa_circ_0110389 suppressed the expression of Beclin-1 and LC3B II/I and increased p62 expression, and these effects was reversed by SORT1 overexpression (Fig. 6C); likewise, overexpression of hsa_circ_0110389 increased the expression of Beclin-1 and LC3B II/I and decreased p62 expression, and these effects was reversed by SORT1 knockdown (Fig. 6D).

Collectively, these findings indicated that hsa_circ_0110389 regulates proliferation, migration and invasion of GC cells through SORT1, and the affected autophagy may partly account for the effect on GC proliferation and metastasis.

### 7. Knockdown of hsa_circ_0110389 inhibits growth of GC tumor in vivo

**Fig. 7:**
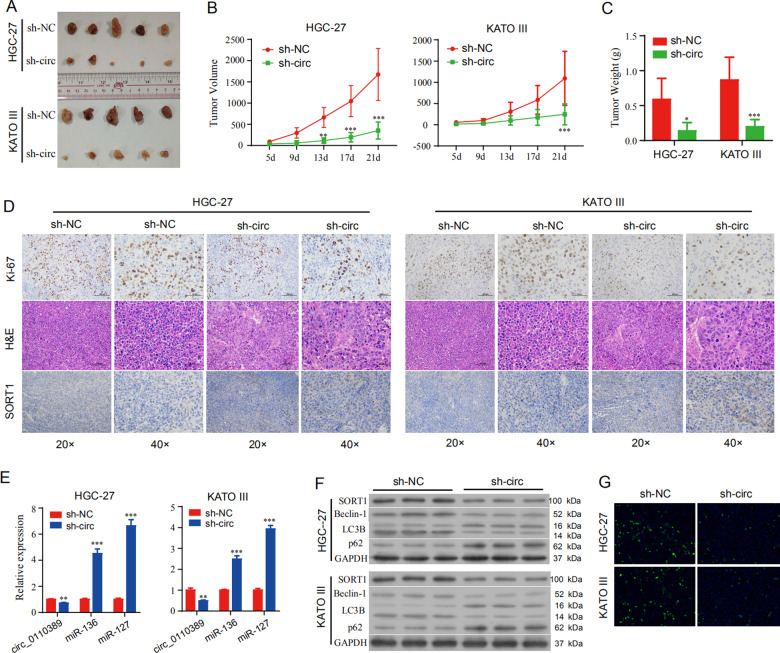
Knockdown of hsa_circ_0110389 inhibits growth of GC tumor in vivo. **A**–**C** Schematic representation (**A**), in vivo growth curve (**B**), and weight at the end points (**C**) of xenograft tumors formed by subcutaneous injection of HGC-27 and KATO III cells stably transfected with sh-NC or sh-circRNA (*n* = 5 for each group). **D** Representative images of H&E staining, Ki-67 staining and IHC for SORT1 of xenograft tumors. **E** The RNA levels of hsa_circ_0110389, miR-127-5p and miR-136-5p in xenograft tumors were detected by qRT-PCR. **F** The protein levels of SORT1 and autophagy-related genes (Beclin-1, LC3B and p62) in xenograft tumors by western blot. **G** The expression of LC3B in xenograft tumors by IF analysis. All data are presented as mean ± SD. *p < 0.05, **p < 0.01, ***p < 0.001.

To determine whether hsa_circ_0110389 affects tumor progression in vivo, we performed tumor growth experiments in NOD/SCID mice bearing both HGC-27 and KATO III cells subcutaneous tumors. As shown, shRNA knockdown of hsa_circ_0110389 significantly decreased the volume and weight of tumors compared to those in the sh-NC group (Fig. 7A-C). And tumors formed in the hsa_circ_0110389 shRNA group exhibited increased inflammatory infiltration and decreased expressing percentage of Ki67 compared with that in the sh-NC group (Fig. 7D). Taken together, knockdown of hsa_circ_0110389 inhibits tumor growth of GC in vivo.

The expressions of hsa-miR-127-5p, hsa-miR-136-5p and SORT1 were also analyzed. qRT-PCR analysis showed that the expression levels of both hsa-miR-127-5p and hsa-miR-136-5p were increased in hsa_circ_0110389 shRNA group (Fig. 7E); both IHC analysis and Western blot results showed that SORT1 expression was impaired by hsa_circ_0110389 silencing (Fig. 7D and F). Moreover, Western blot analysis demonstrated that hsa_circ_0110389 knockdown suppressed the expression of Beclin-1 and LC3B II/I and increased p62 expression (Fig. 7F), which suggested a suppressed autophagy; the suppressed expression of LC3B II/I was also observed by IF assay (Fig. 7G).

## Discussion

With accumulating evidences on the relationship between circRNAs and cancers, quantity of dysregulated circRNAs have been identified and considered as promising potential biomarkers for tumors due to their high stability and specific expression patterns [27, 28]. And although environmental carcinogens have been considered as one of the crucial causes of GC, the function of circRNAs on environmental carcinogens-induced malignant transformation and the molecular mechanisms remain still unclear. In current research, we performed RNA-seq analysis to screen differentially expressed circRNAs in malignantly transformed cells and identified a novel circRNA hsa_circ_0110389 that was significantly upregulated in GC tissues and cells. Meanwhile, hsa_circ_0110389 expression is associated with advanced stages of GC. More importantly, GC patients with high hsa_circ_0110389 expression had significantly shorter OS and DFS, and hsa_circ_0110389 expression level was an independent prognostic factor in GC patients. Therefore, hsa_circ_0110389 may serve as a potential biomarker for GC diagnosis and prognosis.

An increasing number of studies have revealed that circRNAs played important roles in the tumorigenesis and development of human cancers, among which competing endogenous RNA (ceRNA) is one of the most frequently and important mechanism. CircRNAs play as ceRNAs to regulate target gene expression through miRNAs [29, 30]. For example, circPGR was recently found to function as a ceRNA to promote estrogen receptor-positive breast cancer cell growth [31]; our recent study found that hsa_circ_0001829 functions as an oncogene in GC and exerts its effects via miR-155-5p/SMAD2 axis [18]. In current study, results of knockdown and overexpression assays revealed that hsa_circ_0110389 regulates GC cells proliferation, migration and invasion in vitro, and animal studies showed that hsa_circ_0110389 silencing inhibited tumor growth in vivo. Altogether, these results indicated that hsa_circ_0110389 promotes GC progression. Furthermore, we demonstrated that hsa_circ_0110389 was predominantly localized in the cytoplasm and miRNA binding prediction revealed several sponging sites for both miR-127-5p and miR-136-5p in hsa_circ_0110389; we then validated that hsa_circ_0110389 was able to bind to miR-127-5p/miR-136-5p by RNA pull-down and luciferase reporter assays. We also found that miR-127-5p/miR-136-5p expression levels were negatively correlated with the level of hsa_circ_0110389, and miR-127-5p/miR-136-5p could reverse the effect of hsa_circ_0110389 on GC cells proliferation, migration and invasion. Therefore, our results indicated that hsa_circ_0110389 plays its oncogenic role through sponging miR-127-5p and miR-136-5p.

Both miR-127-5p and miR-136-5p have been reported to be significantly downregulated in several types of cancers and to function as tumor suppressors [32–35]. For instances, it has been reported that miR-136-5p is significantly decreased in ovarian serous carcinoma (OSC) tissues and forced expression of miR-136-5p significantly reduced cell growth and migration of OSC cells [34]. In the context of GC, Wang et al. and Guo et al. respectively found that miR-136-5p or miR-127 could serve as a tumor suppressor in GC [32, 35]. In this study, results showed that the expression levels of both miR-127-5p and miR-136-5p were significantly decreased in GC tissues compared with normal tissues, and miR-127-5p/miR-136-5p could reverse the effect of hsa_circ_0110389 on GC cells proliferation, migration and invasion. Therefore, our results are consistent with the notion that miR-127-5p and miR-136-5p function as suppressing factors in GC progression.

As a ceRNA, circRNA can sequester miRNAs to regulate the expression of downstream target genes. Bioinformatics prediction revealed that SORT1 was a potential target of both miR-127-5p and miR-136-5p. Further RNA pull-down and dual luciferase reporter assays demonstrated that miR-127-5p/miR-136-5p could directly bind to the 3′UTR of SORT1. Furthermore, both miR-127-5p and miR-136-5p could regulate the protein expression of SORT1 and simultaneously could affect autophagy. Interestingly, our results indicated that miR-127-5p/miR-136-5p regulates SORT1 expression by repressing translation but not by mRNA degradation. SORT1 gene encodes sortilin protein, a member of the vacuolar protein sorting 10 protein-related family [36]. Aside from its protein-sorting functions, sortilin regulates lipid metabolism and inflammation. Studies in mice showed that hepatic sortilin overexpression reduced the production of Apolipoprotein B (ApoB) [37]; Mechanistically, Amengual et al. found that autophagy is required for SORT1-mediated degradation of ApoB [38]. In addition, sortilin recently emerged as a promising tumor target as its expression was found to be increased in several types of cancers including digestive cancers [39–41], but its expression and impact in GC is unclear. Autophagy is a highly conserved intracellular homeostasis mechanism through which cellular material is delivered to lysosomes for degradation, resulting in the basal turnover of cell components and providing energy and macromolecular precursors. In the world of oncology, autophagy has context-dependent effects: it is known that autophagy inhibits tumor progression, and conversely, once cancer is established, activated autophagic flux often enables cancer cell survival and growth [42]. In our study, we discovered that hsa_circ_0110389 silencing could decrease SORT1 expression; in addition, we found that SORT1 overexpression significantly reversed the hsa_circ_0110389 knockdown induced suppression of proliferation, invasion and migration of GC cells, and SORT1 knockdown significantly blocked the hsa_circ_0110389 overexpression induced promotion of proliferation, invasion and migration in GC cells. Moreover, hsa_circ_0110389 regulated the expression of Beclin-1 and LC3B II/I and p62 expression, and this regulation could be reversed by SORT1, which is consistent with the previous study that SORT1 is a regulator of autophagy [38]. Taken together, these data support our hypothesis that hsa_circ_0110389 acts as a ceRNA to facilitate SORT1-mediated proliferation and metastasis by absorbing miR-127-5p/miR-136-5p in GC, and the affected autophagy by SORT1 may partly account for the effect on GC proliferation and metastasis.

In summary, we identified a novel circRNA hsa_circ_0110389 that was significantly up-regulated in both GC tissues and cell lines, and high hsa_circ_0110389 expression associated with advanced stages of GC and predicts poor prognosis. Furthermore, we demonstrated that hsa_circ_0110389 promoted GC progression by functioning as a sponge for both miR-127-5p and miR-136-5p to upregulate SORT1 expression. Our study firstly identifies the function of hsa_circ_0110389 on GC progression, which provides novel insights for diagnosis/prognosis and therapy for GC.

## Supplementary information

Supplementary table 1

Supplementary material

Figure S1

Figure S2

Figure S3

## Data Availability

The datasets for the current study are available from the corresponding author on reasonable request.
