# Supplementary material for: Circular RNA hsa_circ_0110389 promotes gastric cancer progression through upregulating SORT1 via sponging miR-127-5p and miR-136-5p
Source: Cell Death Dis. 2021 Jun 23;12(7):639. doi: 10.1038/s41419-021-03903-5 (PMC8222372; doi:10.1038/s41419-021-03903-5)
Supplement: Supplementary file 1 — Supplementary table 1 [file 41419_2021_3903_MOESM1_ESM.docx]

**Supplementary table 1** Correlation between hsa_circ_0110389 expression and clinicopathological parameters in gastric cancer (*n* = 110)

| Parameters | Category | No. | hsa_circ_0110389 expression | | χ^2^ | P |
| --- | --- | --- | --- | --- | --- | --- |
|  |  |  | Low | High |  |  |
| Age |  |  |  |  |  |  |
|  | <65 | 67 | 31 | 36 | 0.955 | 0.329 |
|  | ≥65 | 43 | 24 | 19 |  |  |
| Gender |  |  |  |  |  |  |
|  | Male | 63 | 32 | 31 | 0.037 | 0.847 |
|  | Female | 47 | 23 | 24 |  |  |
| Differentiation |  |  |  |  |  |  |
|  | Well | 44 | 28 | 16 | 5.455 | 0.020 |
|  | Moderate + Poor | 66 | 27 | 39 |  |  |
| T stage |  |  |  |  |  |  |
|  | T1+T2 | 34 | 27 | 7 | 17.028 | 0.000 |
|  | T3+T4 | 76 | 28 | 48 |  |  |
| N stage |  |  |  |  |  |  |
|  | N0+N1 | 39 | 37 | 2 | 48.664 | 0.000 |
|  | N2+N3 | 71 | 18 | 53 |  |  |
| M stage |  |  |  |  |  |  |
|  | M0 | 81 | 49 | 32 | 13.533 | 0.000 |
|  | M1 | 29 | 6 | 23 |  |  |
| UICC stage |  |  |  |  |  |  |
|  | I+II | 39 | 33 | 6 | 28.960 | 0.000 |
|  | III+IV | 71 | 22 | 49 |  |  |
| Nerve invasion |  |  |  |  |  |  |
|  | Yes | 60 | 29 | 31 | 0.147 | 0.702 |
|  | No | 50 | 26 | 24 |  |  |
| Vessel invasion |  |  |  |  |  |  |
|  | Yes | 63 | 31 | 32 | 0.037 | 0.847 |
|  | No | 47 | 24 | 23 |  |  |
