# Supplementary material for: Circular RNA hsa_circ_0110389 promotes gastric cancer progression through upregulating SORT1 via sponging miR-127-5p and miR-136-5p
Source: Cell Death Dis. 2021 Jun 23;12(7):639. doi: 10.1038/s41419-021-03903-5 (PMC8222372; doi:10.1038/s41419-021-03903-5)
Supplement: Supplementary file 2 — Supplementary material [file 41419_2021_3903_MOESM2_ESM.docx]

**Supplementary Figure legends**

**Figure S1. Quantification of EdU positive cells and number of colonies.**  **(A, C, E, G, I, K)** HGC-27 and KATO III cells were transfected with sh-NC, sh-circ, sh-circ+inh-NC, sh-circ+inh-127, sh-circ+inh-136, sh-circ+EV or sh-circ+SORT1. Then the ability of cell proliferation and growth was assessed by EdU assay **(A, E, I)** or colony formation assay **(C, G, K).**  **(B, D F, H, J, L)** AGS and NCI-N87 cells were transfected with pCD5-NC, pCD5-circ, pCD5-circ+mimic-NC, pCD5-circ+mimic-127, pCD5-circ+mimic-136, pCD5-circ+shNC or pCD5-circ+shSORT1. Then the ability of cell proliferation and growth was assessed by EdU assay **(B, F, J)** or colony formation assay **(D, H, L).** All data are presented as mean ± SD. EV, empty vector. inh, inhibitor. *vs sh-NC/pCD5-NC group, #vs EV/shNC group. ** p < 0.01, *** p < 0.001, ## p < 0.01, ### p < 0.001.

**Figure S2. Width of cell migration and number of invasive cells. (A, C, E, G)** HGC-27 and KATO III cells were transfected with sh-NC, sh-circ, sh-circ+inh-NC, sh-circ+inh-127, or sh-circ+inh-136. Then the ability of cell migration and invasion was assessed by wound healing **(A, E)** and transwell invasion **(C, G)** assays, respectively. **(B, D, F, H)** AGS and NCI-N87 cells were transfected with pCD5-NC, pCD5-circ, pCD5-circ+mimic-NC, pCD5-circ+mimic-127 or pCD5-circ+mimic-136. Then the ability of cell migration and invasion was assessed by wound healing **(B, F)** and transwell invasion **(D, H)** assays, respectively. All data are presented as mean ± SD. *vs sh-NC/pCD5-NC group, #vs inh-NC/mim-NC group. * p < 0.05, ** p < 0.01, *** p < 0.001, ## p < 0.01, ### p < 0.001.

**Figure S3. Hsa_circ_0110389 promotes the migration and invasion of GC cells through SORT1.** **(A, C, E, G)** HGC-27 and KATO III cells were transfected with sh-NC, sh-circ, sh-circ+EV or sh-circ+SORT1. Then the ability of cell migration and invasion was assessed by wound healing assay **(A, C)** and transwell assay **(E, G).**  **(B, D, F, H)** AGS and NCI-N87 cells were transfected with pCD5-NC, pCD5-circ, pCD5-circ+shNC or pCD5-circ+shSORT1. Then the ability of cell migration and invasion was assessed by wound healing assays **(B, D)** and transwell assay **(F, H).** *vs sh-NC/pCD5-NC group, #vs EV/shNC group. ** p < 0.01, *** p < 0.001, ## p < 0.01, ### p < 0.001.
