# Supplementary figures and images for: Circular RNA hsa_circ_0110389 promotes gastric cancer progression through upregulating SORT1 via sponging miR-127-5p and miR-136-5p
Source: Cell Death Dis. 2021 Jun 23;12(7):639. doi: 10.1038/s41419-021-03903-5 (PMC8222372; doi:10.1038/s41419-021-03903-5)

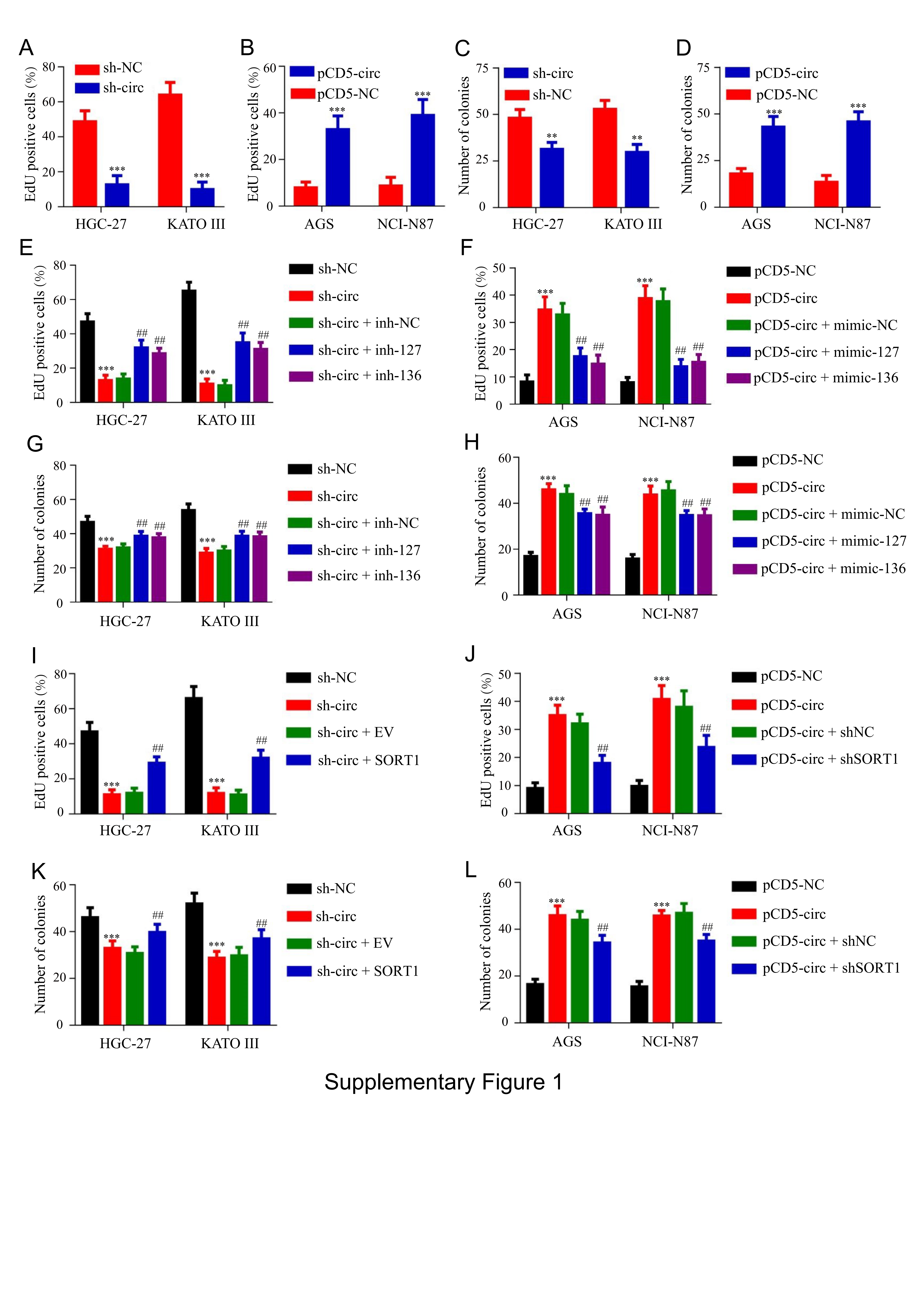

Supplement: Supplementary file 3 — Figure S1 [file 41419_2021_3903_MOESM3_ESM.png]

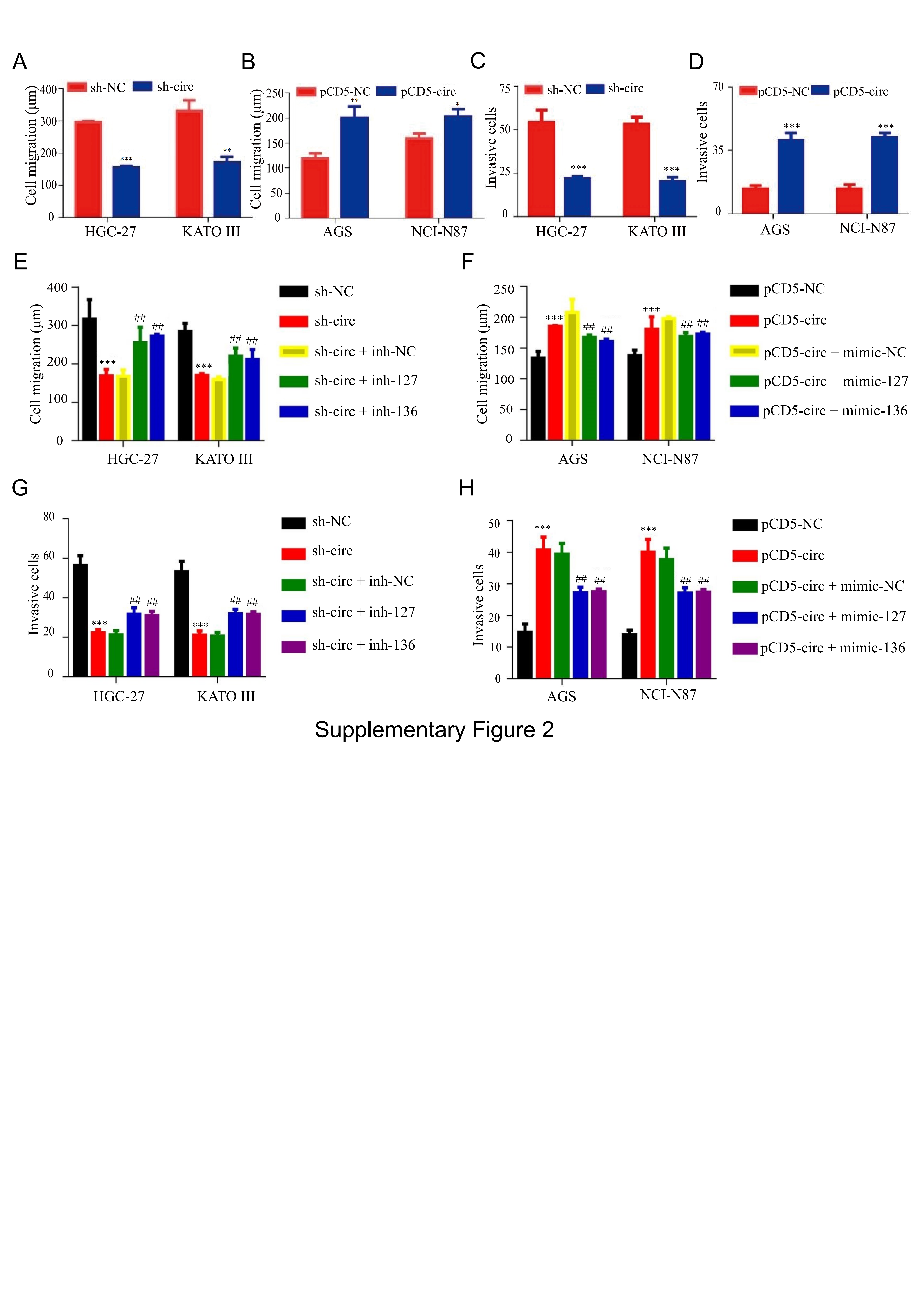

Supplement: Supplementary file 4 — Figure S2 [file 41419_2021_3903_MOESM4_ESM.png]

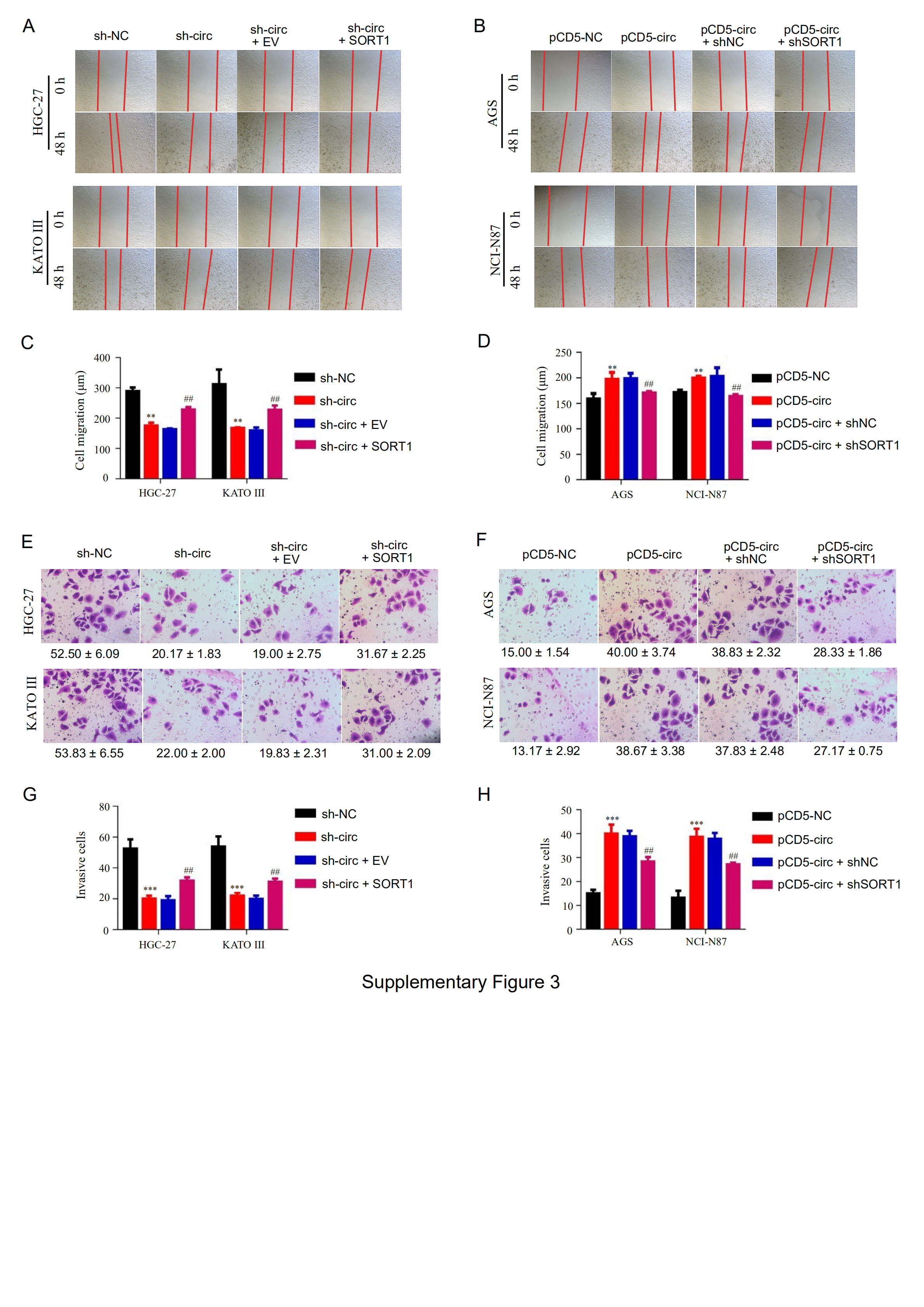

Supplement: Supplementary file 5 — Figure S3 [file 41419_2021_3903_MOESM5_ESM.png]
